# The interplay between vector microbial community and pathogen transmission on the invasive Asian tiger mosquito, *Aedes albopictus*: current knowledge and future directions

**DOI:** 10.3389/fmicb.2023.1208633

**Published:** 2023-07-27

**Authors:** Mario Garrido, Jesús Veiga, Marta Garrigós, Josué Martínez-de la Puente

**Affiliations:** ^1^Department of Parasitology, Faculty of Pharmacy, University of Granada, Granada, Spain; ^2^Ciber de Epidemiología y Salud Pública (CIBERESP), Madrid, Spain

**Keywords:** *Aedes*, bacteria, invasive species, metabarcoding, microbiota, vectors, mosquito-borne pathogens, *Wolbachia*

## Abstract

The invasive Asian tiger mosquito *Aedes albopictus* is nowadays broadly distributed with established populations in all continents except Antarctica. In the invaded areas, this species represents an important nuisance for humans and, more relevant, it is involved in the local transmission of pathogens relevant under a public health perspective. *Aedes albopictus* is a competent vector of parasites such as *Dirofilaria* and viruses including dengue virus, Zika virus, and chikungunya virus, among others. The mosquito microbiota has been identified as one of the major drivers of vector competence, acting upon relevant vector functions as development or immunity. Here, we review the available literature on the interaction between *Ae. albopictus* microbiota and pathogen transmission and identify the knowledge gaps on the topic. Most studies are strictly focused on the interplay between pathogens and *Wolbachia* endosymbiont while studies screening whole microbiota are still scarce but increasing in recent years, supported on Next-generation sequencing tools. Most experimental trials use lab-reared mosquitoes or cell lines, exploring the molecular mechanisms of the microbiota-pathogen interaction. Yet, correlational studies on wild populations are underrepresented. Consequently, we still lack sufficient evidence to reveal whether the microbiota of introduced populations of *Ae. albopictus* differ from those of native populations, or how microbiota is shaped by different environmental and anthropic factors, but especially, how these changes affect the ability of *Ae. albopictus* to transmit pathogens and favor the occurrence of outbreaks in the colonized areas. Finally, we propose future research directions on this research topic.

## Introduction

Emerging infectious diseases have spread dramatically during the last decades, driven by human-induced socio-economic and environmental changes (Morand, 2020; Baker et al., 2021). Different components of global change, such as climate change and landscape anthropization, alter the geographic distribution of pathogens and vectors, increasing the exposure of human and animal populations to new diseases. Global trades and travels provide mosquitoes with routes to spread, facilitating and accelerating their expansion, which promotes the colonization of new areas by diseases (Eritja et al., 2017; Ibáñez-Justicia et al., 2020). This scenario creates uncertainty in disease risk for humans (e.g., Schaffner et al., 2013; Akiner et al., 2016), poses a significant threat to people, and implies a global economic (Diagne et al., 2021) and conservation challenge.

Vector-borne diseases are a global concern, particularly those transmitted by mosquitoes (Culicidae) that serve as the primary vectors of pathogens affecting humans and other animals (Tolle, 2009; WHO, 2020). Approximately 10% of known mosquito species are recognized as potential or confirmed vectors of pathogens with public health relevance (Yee et al., 2022). Mosquitoes of the *Aedes* genus includes numerous vector species of great concern due to their highly invasive character. The yellow fever mosquito (*Aedes aegypti*), the Asian tiger mosquito (*Aedes albopictus*), and, to a lesser extent, the Korean bush mosquito (*Aedes koreicus*) and the rock pool mosquito (*Aedes japonicus*) are representative examples (Cebrián-Camisón et al., 2020). *Aedes aegypti* and *Ae. albopictus* are competent vectors of arboviruses including dengue virus (DENV), chikungunya virus (CHIKV), yellow fever virus (YFV), and Zika virus (ZIKV) (Gutiérrez-López et al., 2023). Although originally from Africa and Asia, respectively, *Ae. aegypti* and *Ae. albopictus* are the most invasive mosquitoes in the world. They have been transported globally through international trade and shipping activities (Reiter and Sprenger, 1987; Powell and Tabachnick, 2013; Laporta et al., 2023), creating novel epidemiological scenarios worldwide. *Aedes albopictus*, in particular, has spread from its native range to, at least, 28 other countries worldwide (ECDC, 2023; Laporta et al., 2023). In Europe, for example, this species was first detected in Albania in 1979 and spread during the 1980s. Nowadays, it is established in more than 15 European countries (ECDC, 2023; Laporta et al., 2023), including all the European Mediterranean Basin. The local abundance of *Ae. albopictus*, coupled with the presence of infected hosts and other factors, has allowed the occurrence of local outbreaks of imported diseases, such as chikungunya fever in Italy and France (Tomasello and Schlagenhauf, 2013; Calba et al., 2017; Venturi et al., 2017), dengue in Croatia, France and Spain (Tomasello and Schlagenhauf, 2013; Delisle et al., 2015; Succo et al., 2016) and Zika in France (Giron et al., 2019). Moreover, *Ae. albopictus* has been implicated in the local transmission of *Dirofilaria* parasites from animal reservoirs to humans in Asia, USA, and Italy (Cancrini et al., 2003, 2007; Gratz, 2004).

The importance of a particular mosquito species for the transmission of pathogens is determined by different components of vector competence (Beerntsen et al., 2000; Stewart Merrill and Johnson, 2020), which refer to the ability of mosquitoes to successfully acquire, maintain, and transmit a pathogen between hosts. Understanding this concept is key to predict the potential of mosquitoes to create new epidemiological scenarios. It is, therefore, crucial to investigate how external factors, such as breeding sites characteristics, and internal factors, such as symbiotic bacteria, affect the vector competence of a particular species in natural ecosystems (Lefèvre et al., 2013; Stewart Merrill and Johnson, 2020). Over the past few decades, researchers have shown that mosquito microbiota plays a critical role in shaping vector competence (Cansado-Utrilla et al., 2021), modulating important functions including the development (Coon et al., 2014, 2016), behavior (Ruiz-Lopez, 2020), digestion and reproduction (Fouda et al., 2001), or physiology and immunity (Dong et al., 2009; Kambris et al., 2010) of insect vectors. Mosquito microbiota has been identified as a key component determining the development of pathogens in the mosquito midgut, as well as mosquito resistance to pathogens and the cost of parasite infections in terms of survival rate [reviewed in Clayton et al. (2014), Gabrieli et al. (2021)]. Consequently, the composition of the microbiota of wild mosquitoes may impact their vector competence and the dynamics of transmission of mosquito-borne pathogens (Boissière et al., 2012; Charan et al., 2013; Souza-Neto et al., 2019). Indeed, previous studies have identified environmental-related differences in the microbiota between mosquito species and populations (Coon et al., 2016; Muturi et al., 2017; Duguma et al., 2019) that could affect their competence (Martínez-de la Puente et al., 2018) and, ultimately, may partially explain the differences in the communities of parasites in host populations.

Based on the significance of mosquito microbiota in the transmission of pathogens, and the relevance of *Ae. albopictus* as a major vector of mosquito-borne pathogens worldwide, the aim of this article is review the role of *Ae. albopictus* microbiota in pathogen transmission. While most recent findings rely on *Ae. aegypti* (see, for example, Ramirez et al., 2012; Monteiro et al., 2019), our knowledge on how microbiota affects pathogen transmission in other species of concern is limited. Given the potential threat posed by the widespread expansion of *Ae. albopictus*, it is crucial to unravel how environmental heterogeneity affects mosquito microbiota and to determine the mechanisms mediating its effects on vector competence (Stewart Merrill and Johnson, 2020) and disease risk in both endemic and invaded areas. Here, we overview the published knowledge on factors shaping microbiota of individual mosquitoes and populations and how impact pathogen transmission, attending also to the molecular methods used in the studies. Given the significant role of certain endosymbionts, mainly *Wolbachia* spp. and *Asaia* spp., in mosquito pathogen transmission (Johnson, 2015; Rosso et al., 2018; Ross et al., 2019), they were also considered in this review. Finally, we identified current knowledge gaps on the topic and suggest future research directions in the area.

## From single-bacteria detection to microbial screening

One of the earliest studies on the tripartite interaction between *Ae. albopictus*, pathogens, and mosquito bacterial communities screened chikungunya (CHIKV)-infected and uninfected mosquitoes for differences in *Wolbachia* abundances (Tortosa et al., 2008). Indeed, a significant amount of publications on the topic aims to determine the presence and/or abundance of certain endosymbionts that may be relevant for pathogen transmission (see Table 1 for examples). *Wolbachia* is the most paradigmatic example, its potential as vector borne diseases suppressor has been discussed at length in recent decades (e.g., Hedges et al., 2008; Hoffmann et al., 2015). Several naturally-occurring *Wolbachia* strains have been identified in a range of species of mosquitoes (Bourtzis et al., 2014; Sicard et al., 2019). Specifically, *Ae. albopictus* is naturally infected by the strains *w*AlbA and *w*AlbB, whose impact on viral infections has been assessed in a fair number of studies, mainly on the viruses CHIKV (Tortosa et al., 2008; Raquin et al., 2015), DENV (dengue virus; Mousson et al., 2012; Huang et al., 2020; Sasaki et al., 2022), and ZIKV (Huang et al., 2020), but also in other pathogens, as entomopathogenic fungi (Ramirez et al., 2021). Molecular techniques, such as PCR and qPCR, have been extensively used in all those studies focused on a single endosymbiotic bacterium. Sometimes in combination with other analytical tools as, for example, the use of taxonomic microarrays (Zouache et al., 2012), immunoassays techniques (McLean et al., 2019), or cell lines cultures (Mousson et al., 2012), usually used in transinfection trials.

**TABLE 1:** Summary of the relevant information contained in the articles most consulted for the review on the interplay between vector microbial community and pathogen transmission on the invasive Asian tiger mosquito, *Aedes albopictus*.

References	Type	Species	wild/Lab Colony/Cell line	Molecular approach	Microbiota/ Endosymbiont	Pathogen/ Parasite	Developmental stage	Tissue	Country/ Region
Calle-Tobón et al., 2022	C	*Ae. aegypti*; *Ae. albopictus*	Wild collected mosquitoes	PCR/qPCR for *Wolbachia*; NGS for virome screening	*Wolbachia*, natural strain; viral microbiota	Pathogenic virus (virome screening)	Adult female mosquitoes	Whole	Medellín, Colombia.
Zhao et al., 2022	E	*Ae. albopictus*	Lab colony, from wild mosquitoes	RT-PCR for DENV; NGS for microbiota	Bacterial microbiota	DENV-2	Adult female mosquitoes	Whole	Guangzhou, China.
Sasaki et al., 2022	E	*Ae. albopictus*	Lab colony, from wild mosquitoes	PCR for *Wolbachia*; Vero cells/plaque assay for DENV	*Wolbachia*, *w*AlbA and *w*AlbB strains	DENV-1, DENV-2, DENV-3	Adult female mosquitoes	Salivary glands, midgut, and carcasses	Japan (3 sites).
Onyango et al., 2021	E	*Ae. albopictus*	F15 lab colony, from wild mosquitoes	RT-qPCR for ZIKV; NGS for microbiota	Bacterial microbiota, focus on *E. anophelis*	ZIKV (lab strain HND 2016–19563)	Adult female mosquitoes	Gut (infective.), legs (dissemin.), saliva (transmiss.)	NY, USA.
Ramirez et al., 2021	E	*Ae. albopictus*, *Cx. pipiens*	Individuals from wild collected eggs	PCR for *Wolbachia*;	*Wolbachia*, *w*AlbA and *w*AlbB strains	*Beauveria bassiana* (MBC076); *Beauveria brongniartii* (MBC397).	Adult female mosquitoes	Whole	From Univ. Washington, MO, USA.
Pereira et al., 2021	E/C	*Ae. albopictus*	Individuals from wild collected eggs	PCR/RT-PCR for *Wolbachia* and DENV; NGS for microbiota	Bacterial microbiota; *Wolbachia*, *w*AlbA and *w*AlbB strains	MAYV	Adult female mosquitoes	Abdomen and head-torax separated	Brazil (3 sites).
Seabourn et al., 2020	C	*Ae. albopictus*	Wild collected mosquitoes	PCR for *A. taiwanensis*; NGS for microbiota	Bacterial microbiota; *Asaia*; *Wolbachia*, *w*AlbA and *w*AlbB strains	*Ascogregarine taiwanensis*	Adult female and male mosquitoes	Whole	Maui, Hawaii, USA (8 sites).
Huang et al., 2020	C	*Ae. albopictus*	Wild collected mosquitoes	PCR/RT-PCR for *Wolbachia*, ZIKV, and DENV;	*Wolbachia*, *w*AlbA and *w*AlbB strains	DENV and ZIKV natural infections	Immature and adult mosquitoes	Whole	Hong Kong (57 sites).
Bhattacharya et al., 2020	E	*Ae. albopictus*	Mosquito cell lines	Cell lines infected/not with *Wolbachia*; RT-PCR for SINV, CHIKV, and ZIKV	*Wolbachia*, *w*Mel, *w*Stri, *w*AlbB strains	SINV-nLuc, CHIKV18125, and ZIKV MR766	-	-	-
Mancini et al., 2020	E	*Ae. albopictus*	Lab colony (>F20), from wild mosquitoes	PCR/RT-PCR for *Wolbachia*, DENV, and ZIKV	*Wolbachia*, *w*Au strain	DENV-2- C Strain and ZIKV-MP1751	Adult female mosquitoes	Whole	Kuala Lumpur, Malaysia.
Ekwudu et al., 2020	E	*Ae. albopictus*	Mosquito cell lines	Immunofocus for DENV; plaque assays for other viruses.	*Wolbachia*, *w*AlbB strain	WNV-KUN, RRV, BFV, SINV, DENV-2, DENV-3, ZIKV strains.	-	-	-
McLean et al., 2019	E	*Ae. albopictus*	Mosquito cell lines	Cell lines infected/not with *Wolbachia*; RT-PCR and ELISA for CFAV and PCLV	*Wolbachia*, *w*Mel or *w*MelPop-CLA	CFLV and PCLV, undefined strains	-	-	-
Teramoto et al., 2019	E	*Ae. albopictus*	Mosquito cell lines	Plaque assay and RT-qPCR for DENV2	*Wolbachia*, *w*MelPop-CLA	DENV-2	-	-	-
Schultz et al., 2018	E	*Ae. albopictus*	Mosquito cell lines	Immunofocus plaque assays for DENV.	*Wolbachia*, *w*Stri *strain*	CHIKV-131/25, LACV, DENV-2, YFV-17-D, ZIKV-PRVABC59	-	-	-
Schultz et al., 2017	E	*Ae. albopictus*	Mosquito cell lines	Cell lines infected/not with *Wolbachia*; FFU assays for ZIKV	*Wolbachia*, *w*Mel, *w*Stri, *w*AlbB strains	African strain ZIKV MR766	-	-	-
Duchemin et al., 2017	E	5 local *Aedes/Culex* spp.; *Ae. albopictus* (invasive spp.)	F4/F9 Lab colony, from wild collected eggs	PCR/RT-PCR for *Wolbachia* and ZIKV	*Wolbachia*, undefined strain	ZIKV Cambodia 2010 strain	Adult female mosquitoes	Whole (infectivity) and salivary glands (transmission)	Hammond Island, Australia.
Charan et al., 2016	C	*Ae. albopictus, Ae. aegypti, Ae. vittatus*	Wild collected mosquitoes	Plate culture/PCR for bacteria identification;	Bacterial community	DENV undefined natural strain,	Adult female mosquitoes	Midgut	Rajasthan, India.
Raquin et al., 2015	E	*Ae. albopictus*	Mosquito cell lines	PCR/RT-PCR for *Wolbachia* and CHIKV;	*Wolbachia (w*AlbB*)*	CHIKV 06.21 strain	-	-	-
Mousson et al., 2012	E	*Ae. albopictus*	F2 lab colony, from wild mosquitoes	qPCR/RT-qPCR for *Wolbachia* and DENV; FFU assay for DENV in saliva	*Wolbachia (w*AlbA *and w*AlbB*)*	DENV, DENV-2 provided by Prof. Leon Rosen	Adult female mosquitoes	Midgut (infectivity), legs (dissemination), and salivary glands (transmission)	La Reunion Island, France.
Zouache et al., 2012	E	*Ae. albopictus*	F2 lab colony, from wild mosquitoes	Taxonomic microarrays/qPCR for endosymbionts; RT-qPCR for CHIKV	Bacterial microbiota, diversity of endosymbionts	CHIKV-E1-226V	Adult female mosquitoes	Whole	La Reunion Island, France.
Tortosa et al., 2008	E	*Ae. albopictus*	F4 lab colony, from wild mosquitoes	PCR/qPCR for *Wolbachia*;	*Wolbachia w*AlbA and *w*AlbB strains	CHIKV-E1-226V	Adult female mosquitoes	Whole	La Reunion Island, France.

E, experimental; C, correlational.

Besides natural strains, transfection of *Wolbachia* among insect species has been suggested as an efficient mechanism to block pathogen transmission in vectors (e.g., Hedges et al., 2008; Hoffmann et al., 2015). Viral infections are limited in *Ae. albopictus* mosquito cell lines transinfected with the *w*Mel and *w*MelPop strains from *Drosophila melanogaster* (McLean et al., 2019; Teramoto et al., 2019; Bhattacharya et al., 2020), the *w*Au from *Drosophila simulans* (Mancini et al., 2020), or the *w*Stri isolated from the planthopper *Laodelphax striatellus* (Schultz et al., 2017, 2018; Bhattacharya et al., 2020). The potential of other endosymbionts, as *Asaia* or *Pantoea*, as efficaciously suppressors of vector borne diseases has also been proposed (Guégan et al., 2018b), although we lack specific studies deepening on their effect on *Ae. albopictus*’ vector competence (but see, for example, Rosso et al., 2018; Seabourn et al., 2020).

The screening of microbial communities has revealed as a practical tool to detect new insect symbionts potentially involved in pathogen transmission. The development of novel molecular techniques, together with the reduction of their costs, have popularized these studies in the last years. New high-throughput sequencing techniques, as next-generation sequencing (NGS), have been used to characterize *Ae. albopictus* microbiota in relation to its vector competence (Seabourn et al., 2020; Onyango et al., 2021; Zhao et al., 2022). Most studies are aimed to unveil the role of vector microbial communities on ZIKV and DENV transmission (e.g., Duchemin et al., 2017; Schultz et al., 2018; Huang et al., 2020; Mancini et al., 2020). Complementary, these techniques have been applied to detect how microbiota variations of mosquitoes with different statuses, such as food regime, correlate with infections by ZIKV (Onyango et al., 2021), MAYV (Pereira et al., 2021), or DENV (Zhao et al., 2022). In addition, whole microbiota has been screened by Seabourn et al. (2020) using NGS, despite being focused on *w*AlbA and *w*AlbB and the infection by *A. taiwanensis*, while Calle-Tobón et al. (2022) do it to screen whole viromes.

## Assessment of vector competence: experimental and correlational studies

Strictly, vector competence should be assessed using specific trials to discriminate from alternative mechanisms or findings (see, for example, Stewart Merrill and Johnson, 2020), for example, estimating the infection success, dissemination, and transmission rates of pathogens in mosquitoes (see, for example, Gutiérrez-López et al., 2019). Those criteria have been applied to assess *Ae. albopictus’* vector competence in relation to *Wolbachia* and, to a lesser extent, to the whole microbiota (Table 1). Good example of the latter is the experimental trial performed by Onyango et al. (2021) where the effect of ZIKV infection and temperature regimes on microbiota composition was evaluated in three experimental groups: individuals fed only with 10% sucrose, and those fed with infected- and uninfected-blood. Results were consistent with those of Muturi et al. (2016), compared to sucrose-fed individuals, that is mosquitoes fed with blood showed a significant reduction in the microbiota diversity, being more pronounced in those fed with ZIKV-infected blood meals. Moreover, Onyango et al. (2021) observed a negative correlation between ZIKV and the *Elizabethkingia anophelis albopictus* strain, as well as a reduction in ZIKV infection when mosquitoes were fed with a supplemental diet of *E. anophelis aegypti*.

Following a similar approach, Pereira et al. (2021) studied the impact of certain *Wolbachia* strains on the vector potential of *Ae. albopictus* for Mayaro virus (MAYV). Adult females were fed with MAYV-infected blood and, several days after, infection and dissemination were assessed by screening head and thorax samples for the presence of the virus. Subsequently, transmission was tested infecting naïve *Ae. aegypti* individuals with the saliva of MAYV-infected *Ae. albopictus*. Results from the analyses of head and thorax tissues suggested that *Wolbachia w*AlbB inhibits MAYV, indicating a possible effect on pathogen transmission. In other trials, neither Duchemin et al. (2017), working with Australian-native *Ae. albopictus* population, nor Sasaki et al. (2022), comparing native and non-native populations, detected an effect of *Wolbachia* on ZIKV and DENV transmission, respectively, but infection/dissemination were experimentally proved. Yet, Mousson et al. (2012) demonstrated that *Wolbachia* limits DENV transmission, estimated as the virus load in the mosquito salivary glands, but not oral infection or viral replication (dissemination). For *Ae. albopictus*, *Wolbachia* transinfection from *Drosophila simulans* (*w*Au strain) has been proved to effectively blocks ZIKV infection (Mancini et al., 2020).

Beyond the mentioned experimental approaches, valuable information on *Ae. albopictus* pathogens transmission can be extracted from other studies not directly assessing vector competence but other related components. Zhao et al. (2022) detected changes in the microbiota composition of female mosquitoes before and after DENV experimental infection, identifying those bacterial groups whose abundance varied significantly (e.g., *Neurospora crassa*, *Gammaproteobacteria* spp., and *Lactobacillus harbinensis*). Moreover, authors identified certain microorganisms, such as *Alphaproteobacteria*, that may be involved in the DENV susceptibility of *Ae. albopictus*, as they may affect immunity and/or metabolism of mosquitoes. The abundance of *Alphaproteobacteria* and *Gammaproteobacteria*, as well as other groups such as the *Enterobacteriaceae* family, also varied in response to oral CHIKV infections (Zouache et al., 2012). A specially relevant finding is the reduction of endosymbiont abundance, such as *Wolbachia*, which suggests the existence of competition mechanisms between the virus and the endosymbiont (Hawlena et al., 2022). Studies on single endosymbionts also supported this hypothesis: a slight reduction in the density of *Wolbachia* in mosquitoes was observed upon CHIKV experimental infection (Tortosa et al., 2008). Complementary, *Wolbachia* clearance from natural-infected mosquitoes using antibiotics did not affect their susceptibility to fungal entomopathogens, but did affect fungi abundance and some gene expression patterns related to defense against fungal infections (Ramirez et al., 2021).

Cell culture techniques have also contributed to disentangling the mechanisms by which endosymbionts act as suppressors of pathogenic agents (examples in Table 1). Support for the resource-competition theory between *Wolbachia* and certain pathogens is also provided from cell-lines experiments. In different mosquito cell lines (e.g., C710, C/*w*Stri1, Aa23, C6/36 cells) stably-infected with the *Wolbachia w*AlbB and *wStri* (transinfected from *Laodelphax striatellus*) strains, LaCrosse virus (LCV) or vesicular stomatitis virus (VSV) infections were unaffected, while infections caused by DENV, CHIKV, ZIKV, and yellow fever viruses were constrained (Schultz et al., 2017, 2018). However, cholesterol supplementation allows *Wolbachia* and ZIKV to grow together. Moreover, Bhattacharya et al. (2020) provided molecular insights on how *Wolbachia* (strains *w*AlbB, *w*Stri, and *w*Mel from *Drosophila melanogaster*) inhibits the early stages of infections by different *Flaviviridae* and *Togaviridae* viruses, including ZIKV, CHIKV, and Sindbis virus (SINV) in the cell line RML-12. Similarly, McLean et al. (2019) detected no replication of the flavivirus cell-fusing agent virus (CFAV) in RML-12 *Wolbachia*-infected cell lines (with the strains *w*Mel and *w*MelPop-CLA from *Drosophila melanogaster*), nor even in the *Ae. albopictus* (C6/36) cell line (which lacks functional antiviral RNAi response) but did for the Phasi Charoen-like virus (PCLV). The replication, assembly, and secretion of CHIKV were also restricted in mosquito C6/36 cell line in presence of the *Wolbachia w*AlbB strain (Raquin et al., 2015), as occurs for other *Flavivirus* and *Alphavirus* (Ekwudu et al., 2020), or with DENV-type2 in the presence of the pathogenic strain *w*MelPop (Teramoto et al., 2019) using the same cell line.

Correlational studies must definitively contribute to our understanding of the interplay among bacterial communities and pathogen transmission. At early research stages, correlations can provide inputs that must be lately disentangled under experimental conditions. For example, a survey of different natural populations on the island of Maui, Hawai‘I, revealed that bacterial microbiota varied according to geographic location but also to infection by *Ascogregarina taiwanensis*, a protozoan parasite of mosquitoes (Seabourn et al., 2020). Significant differences in midgut bacterial communities of *Ae. albopictus* (as well as *Ae. aegypti* and *Aedes vittatus*) were also detected in different populations from dengue endemic and non-endemic areas in India (Charan et al., 2016). In Hong Kong metropolitan area, a study failed to detect the presence of DENV and ZIKV in *Ae. albopictus*, while *Wolbachia* was stably present in 80% of the samples analyzed (Huang et al., 2020). Another survey in Colombia examined the virome composition in relation to the presence of *Wolbachia* in field-caught mosquitoes (Calle-Tobón et al., 2022) results showed that *Wolbachia* was unrelated with any significant change in the virome richness, diversity, or abundance.

## Target pathogens studied

*Aedes albopictus* is a known vector of emerging and re-emerging arboviruses of special concern (Gutiérrez-López et al., 2023), mostly the ZIKV, DENV, CHIKV arboviruses that produced outbreaks in several countries worldwide, raising an unprecedent number of clinical cases (Fritzell et al., 2018). This is reflected in the available literature (see Table 1 for examples): a growing number of publications on the role of *Ae. albopictus* microbiota in the transmission of ZIKV and DENV were published following the 2015–2016 Zika epidemic (WHO, 2022) and the 2019 dengue outbreaks (WHO, 2019), respectively. The interplay between the *Wolbachia* and vector competence for ZIKV has been explored for different strains: Cambodia 2010, African MR766, and Puerto Rico PRVABC59 (Duchemin et al., 2017; Schultz et al., 2017, 2018). Apart from the above mentioned study on DENV-2 New Guinea strains (Mancini et al., 2020), other works focused exclusively on the DENV-2 strain, the one causing the more severe health cases in humans, confirmed the capacity of *Wolbachia* to limit its infection (e.g., Mousson et al., 2012; Teramoto et al., 2019). Differential susceptibilities to the three DENV serotypes (DENV1, DENV2, and DENV3) were found among native and non-native *Ae. albopictus* populations, naturally infected with *Wolbachia*-infected (Sasaki et al., 2022). However, it is important to clarify that the different virus strains are not equally represented, although they may respond differential to vector microbiota composition. The work of Teramoto et al. (2019) constitutes a good example since they detected differences in the mosquito susceptibility to infection by three strains of DENV. In. addition, inconclusive results have been found for natural undefined DENV strains (Huang et al., 2020), or among dengue-endemic and non-endemic natural areas (Charan et al., 2016). Further studies have simultaneously assessed the effects of *Wolbachia* on ZIKV and DENV natural strains (Huang et al., 2020), on ZIKV strain MP1751 and DENV-2 New Guinea C-strain (Mancini et al., 2020), on ZIKV (MR766 Uganda Strain) with SINV-nLuc and CHIKV18125-capsid-mKate (Bhattacharya et al., 2020), and, on three different ZIKV strains (the Brazilian KU365780, the French Polynesian H/PF/2013, and African MR766 strains) together with other arbovirus strains: DENV-2 ET300, WNV-KUN (MRM 16), RRV (T48), BFV (16313), and SINV-MRM39 (Ekwudu et al., 2020). More recently, Onyango et al. (2021) screened the entire bacterial community of *Ae. albopictus* mosquitoes, evaluating its effect on vector competence for the ZIKV lab strain HND 2016–19563.

Studies on the impact of *Ae. albopictus* microbiota on the development of CHIKV have also been conducted. The E1-226V CHIKV variant has been used to test the responses to infection of both, the microbiota (Zouache et al., 2012) and *Wolbachia* natural strains (Tortosa et al., 2008). The role of endosymbionts as pathogen blockers has been also tested against the CHIKV 06.21 (Raquin et al., 2015) and the CHIKV 131/25 strains (Schultz et al., 2018). Lastly, CHIKV18125-capsid-mKate infectivity was tested in the presence of *Wolbachia* (Bhattacharya et al., 2020), along with that of ZIKV and Sindbis nLuc reporter (SINV-nLuc) viruses.

Further research has explored the impact of *Ae. albopictus* microbiota in the transmission of other pathogens such as Mayaro virus (MAYV; Pereira et al., 2021), *Ascogregarina taiwanensis* (Seabourn et al., 2020), the entomopathogenic fungi *Beauveria bassiana* (MBC076) and *Beauveria brongniartii* (MBC397) (Ramirez et al., 2021), and the Cell-fusing agent virus (CFLV) and Phasi Charoen-like virus (PCLV) (McLean et al., 2019). Calle-Tobón et al. (2022) screened the mosquito virome looking for pathogenic viruses, despite not find any, pathogen screening can be integrated as a feasible solution to not be limited at detecting pathogens of a specific group.

## Mosquitoes’ geographical origin

Originally from Asia, *Ae. albopictus* is currently one of the most invasive mosquito species in the world (ECDC, 2023; Laporta et al., 2023), facing new environmental conditions of colonized areas, which may affect its microbiota and, potentially, its vector competence (e.g., Minard et al., 2015; Coon et al., 2016; Muturi et al., 2017; Duguma et al., 2019). Recent studies have identified simplified microbiota in *Ae. albopictus* from the invaded areas with respect to those of the native distribution range. The comparison of the microbiota of *Ae. albopictus* from Vietnam (native area) and France (invaded populations) revealed a lower bacterial diversity in invaded areas (Minard et al., 2015). Similarly, mosquitoes from Italy showed a lower diversity and a different composition of bacteria than those from the native area (Rosso et al., 2018). Apart from the studies focused on bacterial microbiota, a complementary article compared the fungal microbiota (mycobiome) of native versus introduced populations (Luis et al., 2019). These differences in the composition of the mosquito microbiota may impact their ability for the transmission of pathogens under natural conditions, as suggested by the reported variations in mosquito microbiota of wild populations between dengue-endemic and not endemic areas within its natural area of distribution (e.g., Charan et al., 2016; Huang et al., 2020, Hong Kong and India, respectively) or those in the fungal microbiota populations exposed to different environmental conditions (Tawidian et al., 2021). Additional surveys were carried out in colonized countries, including two in South America (Colombia and Brazil) (Pereira et al., 2021; Calle-Tobón et al., 2022, respectively), and in the Pacific island of Maui (USA) (Seabourn et al., 2020), but these studies did not compared their results from native populations of the species.

In addition, different studies have identified the composition of the microbiota of laboratory colonies established from wild eggs, larvae, or adult *Ae. albopictus* mosquitoes collected, among other places, in China (Zhao et al., 2022), Japan (Mancini et al., 2020; Sasaki et al., 2022), Malaysia, the island of La Reunión (Tortosa et al., 2008; Mousson et al., 2012; Zouache et al., 2012), continental USA (Onyango et al., 2021), or from north Australia (Duchemin et al., 2017). However, *Ae. albopictus* microbiota may differ between field collected mosquitoes and those reared in the laboratory (Baltar et al., 2023) but also between generations within a laboratory colony or wild collected individuals (Hegde et al., 2015), potentially limiting the conclusions obtained about the potential effects of microbiota composition on pathogen development.

In addition to the differential composition of *Ae. albopictus* microbiota between geographically distant areas, mosquito microbiota may largely vary at a local scale. Temporal variation in the microbiota of *Ae. albopictus* have been reported (Saab et al., 2020). Furthermore, different human activities, such as deforestation or urbanization, may alter the mosquito-pathogen relationships (Ferraguti et al., 2016), this is especially relevant for highly anthropogenic species as *Ae. albopictus*. For example, Thongsripong et al. (2018) found a richer bacterial microbiota of *Ae. aegypti* in rural than in suburban habitats. The use of insecticides or antibiotics have been found to affect the bacterial community of *Ae. albopictus* (Guégan et al., 2018a; Juma et al., 2020), modifying the abundance of bacteria with a known role on pathogen transmission including *Elizabethkingia* spp. and *Wolbachia* spp. Yet, the direct impact of anthropogenic stressor on vector competence and pathogen transmission remains to be measured for *Ae. albopictus*.

## Conclusion and future research directions

As far as we know, this is the first review on vector’s microbial community and pathogen transmission on *Ae. albopictus*. Together with *Ae. aegypti*, the Asian tiger mosquito is one of the species spreading faster and more globally in the last decades (ECDC, 2023; Laporta et al., 2023) representing a public health concern. Thus, the information contained in this review provides valuable information for the tracking, control, and prevention of arboviruses transmitted by this mosquito species.

The number of publications on this research topic has increased during the last years, reflecting the growing interest in understanding how disease transmission is shaped by the vector-pathogen-environment interplay. However, the simultaneous study of all components of this tripartite interaction has been traditionally neglected. Interestingly, authors such as Onyango et al. (2021) studied vector competence, experimental infection, and bacteriome response of mosquitoes at same time. However, almost all screened publications can be divided into three main categories: (i) those correlating infection patterns with the presence of *Wolbachia* or, alternatively, to other groups in mosquitoes collected in wild populations, (ii) those detecting changes in the microbiota/endosymbionts of mosquitoes related to geographical locations or sites with differential infection rates by certain pathogens, and (iii) those experimentally assessing the endosymbiont/microbiota-pathogen in cell lines or laboratory colonies. Nonetheless, their contribution to our knowledge on the interactions between mosquitoes, microbiota and pathogen transmission has been crucial. For example, there is accumulate correlative evidence showing a negative relationship between *Wolbachia* strains and certain pathogens as DENV or ZIKV. Yet, there is limited evidence to reveal whether introduced populations of *Ae. albopictus* harbor the same, different, or a simplified microbiota than mosquitoes from native populations (but see Minard et al., 2015), as well as those with different origins, but especially, how these changes affect their vector potential affecting the occurrence of outbreaks in the colonized areas. In addition, further studies should explore the impact of the different components of the global change scenario as environmental drivers impacting bacterial communities and pathogen transmission in natural *Ae. albopictus* populations. This is specially the case of landscape anthropization which affect the exposure to antibiotics and other pollutants in surface water, among other factors, may impact the microbiota composition of mosquitoes and, therefore, their interactions with pathogens. In this respect, the importance of mosquito microbiota in the transmission of *Ae. albopictus* of different pathogens should be addressed for a number of pathogens that are able to transmit including the zoonotic *Dirofilaria* for which, to our knowledge, we lack basic information on the relevance of *Ae. albopictus* microbiota in their transmission.

## Author contributions

JM-P and MGarrido conceived the original idea. MGarrido wrote the first original draft of the manuscript and subsequent versions with considerable assistance from JM-P, JV, and MGarrigós. All authors contributed to manuscript revision.
